# Lipids in cancer: a global view of the contribution of lipid pathways to metastatic formation and treatment resistance

**DOI:** 10.1038/s41389-022-00420-8

**Published:** 2022-08-09

**Authors:** Sophie Vasseur, Fabienne Guillaumond

**Affiliations:** Centre de Recherche en Cancérologie de Marseille, INSERM, Aix-Marseille Université, CNRS, Institut Paoli-Calmettes, F-13009 Marseille, France

**Keywords:** Cancer metabolism, Cancer microenvironment

## Abstract

Lipids are essential constituents for malignant tumors, as they are absolutely required for tumor growth and dissemination. Provided by the tumor microenvironment (TME) or by cancer cells themselves through activation of de novo synthesis pathways, they orchestrate a large variety of pro-tumorigenic functions. Importantly, TME cells, especially immune cells, cancer-associated fibroblasts (CAFs) and cancer-associated adipocytes (CAAs), are also prone to changes in their lipid content, which hinder or promote tumor aggressiveness. In this review, we address the significant findings for lipid contribution in tumor progression towards a metastatic disease and in the poor response to therapeutic treatments. We also highlight the benefits of targeting lipid pathways in preclinical models to slow down metastasis development and overcome chemo-and immunotherapy resistance.

## Introduction

Lipids, as proteins and nucleic acids, make up the building blocks of living cells, serve as important energy sources or intra- or extracellular signaling molecules [1, 2]. During cancer development and progression, cancer cells need to constantly adapt metabolically to tumor microenvironment (TME) evolutions, such as limited vascularization and nutrient bioavailability, as well as increased extracellular matrix (ECM), immune infiltrate and cancer-associated fibroblasts (CAFs) expansion, in order to grow, proliferate and form metastases [3, 4]. Hence, depending on tissue of origin, oncogenic drivers and exogenous nutrient availability, cancer cells may rely on lipid uptake or on intracellular lipid pools, resulting from de novo synthesis, lipid droplets (LD) mobilization (e.g., lipolysis, cholesterol de-esterification), or membrane remodeling [5–8]. Moreover, in TME-rich tumors, recruitment of non-malignant host cells, such as immunosuppressive cells, CAFs, cancer-associated adipocytes (CAAs), can be induced by tumor- or stromal-derived lipid mediators (e.g., Prostaglandin E2 (PGE_2_), fatty acids (FAs), cholesterol) and the acquisition of pro-tumoral functions of TME’s cells depends in part of lipid reprogramming [9–11]. Therefore, having an overview of lipid functions in malignant and non-malignant compartments, and in the dialog between both is absolutely needed before considering systemic treatment targeting lipid metabolism as therapeutic strategies for cancer. Moreover, there are growing evidences from preclinical cancer studies that activated lipid metabolic pathways in tumoral and/or TME compartments participate to the increased cancer cell resistance to chemotherapeutic agents or immunotherapy [9, 12]. Therefore, the targeting of lipid metabolism could also be a promising therapeutic approach to overcome tumor resistance to most common treatments.

In this review, we summarize the main findings showing how lipid and cholesterol metabolism in the tumor and stromal compartments supports metastases formation. As their respective role in parental tumor growth has been stated in several recent reviews [4, 6–8, 13], this aspect will not be addressed in the review. Here, we discuss the role of tumor-derived lipids, as signaling molecules promoting pro-tumoral TME, and how the contribution of TME-specific lipid reprogramming is beneficial for the tumors. Finally, we summarize therapeutic strategies in which targeting lipid pathways can improve tumor response to chemo- or immunotherapy.

### Targeting lipid metabolism to slow down metastatic disease

Metastases for all solid tumors account for 66.7% of cancer deaths [14], and their formation is a multistep process starting at the primary site, then pursuing in the vasculature to finally ending in distant organs [15]. Primary cancer cells need to increase their motility and invasive capacities, through the ECM, to reach the local vasculature and then the circulation [15]. This is accompanied by an epithelial to mesenchymal transition (EMT), during which epithelial cells acquire a mesenchymal phenotype which is prone to colonization of distant sites. However, our understanding of the metabolic changes characterizing each step ultimately leading to the colonization of distant healthy tissues is still far behind that of parental primary tumor [16]. Moreover, according to the metastatic site, cancer cells have also to cope with changes in nutrient and oxygen intakes to survive and expand [16]. Hence, changes in their metabolic program, as well as increased metabolite exchanges with the surrounding non-malignant cells are key determinants of metastatic colonization and growth [17].

#### Role of fatty acid (FA) transport in the metastatic process

**Fig. 1:**
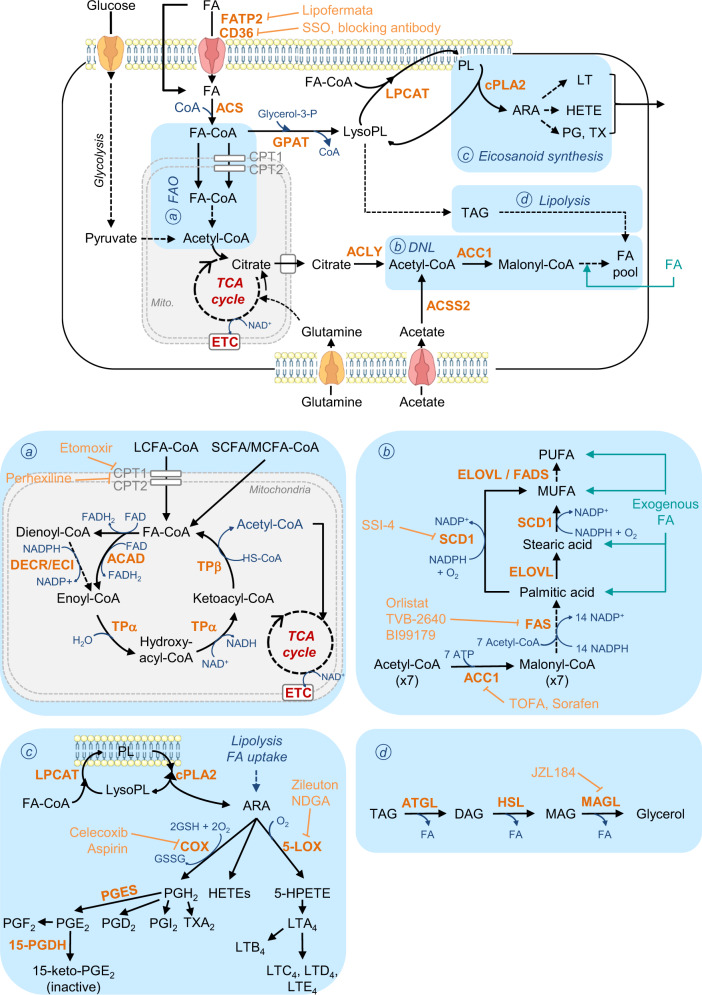
Non-exhaustive representation of lipid metabolic pathways addressed in the review. Fatty acids (FAs), taken up from tumor microenvironment through FA translocase (CD36), FA transport protein (FATP) or by other mechanisms (e.g., passive diffusion, lipoprotein endocytosis), are esterified by acyl-CoA synthetases (ACS) into fatty acyl-CoA (FA-CoA). These latter are used either to build structural or storage lipids or as energy sources through the fatty acid β-oxidation (FAO) pathway. **a** Fatty acid β-oxidation. The first and rate-limiting step of long-chain acyl-CoAs (LCFA-CoAs) oxidation is accomplished by the carnitine palmitoyltransferase 1 (CPT1) located in the outer mitochondrial membrane. CPT1 converts LCFA-CoA into LCFA-carnitine, a necessary step to provide the LCFA-CoA entry into the mitochondrial matrix, whereas short- and medium-chain FA-CoAs (S- and MCFA-CoAs)) can freely diffuse through the mitochondrial inner membrane. Then, a carnitine/acylcarnitine translocase shuttles the LCFA-carnitine across the inner mitochondrial membrane where they are converted back to LCFA-CoAs by CPT2. Unlike saturated FA-CoA, polyunsaturated FA-CoAs (PUFA-CoAs) need to be sequentially converted in trans-2-enoyl-CoA by the 2,4-dienoyl-CoA reductase (DECR) and the Δ3, Δ2-enoyl-CoA isomerase (ECI) enzymes, before to be completely degraded through the β-oxidation cycle. The latter, through the successive action of the acyl-CoA dehydrogenase (ACAD) and the α and β-subunits of trifunctional protein TP, gives rise to acetyl-CoA molecules, which are further oxidized through the tricarboxylic acid (TCA) cycle to generate ATP and the reducing equivalents NADH and FADH_2_. **b** De novo lipogenesis (DNL). Citrate, derived from the TCA cycle, is converted to acetyl-CoA into the cytoplasm by the ATP-citrate lyase (ACLY). Then, acetyl-CoA is carboxylated by the cytoplasmic acetyl-CoA carboxylase 1 (ACC1) into malonyl-CoA, which is considered as the rate-limiting reaction. The next step is catalyzed by the fatty acid synthase (FAS), that synthesizes one molecule of palmitic acid from 7 molecules of malonyl-CoA. Further elongation and insertion of double bonds in carbon chains from FA (provided by DNL and/or diet), are carried out by FA elongases (ELOVLs) and stearoyl-CoA or FA desaturases (SCDs or FADSs), respectively. Glutamine and acetate are also suppliers of citrate and acetyl-CoA useful for DNL. **c** Eicosanoid synthesis. The arachidonic acid (ARA), released from membrane phospholipid (PL) hydrolysis by the cytoplasmic phospholipase A2α (cPLA2α), is further metabolized into either prostaglandins (PG) and thromboxanes (TX), or leukotrienes (LT) and hydroxyeicosatetranoic acids (HETE). Cyclooxygenases (COX) and lipoxygenases (LOX) constitute the respective rate-limiting enzymes of PG and LT/HETE synthesis, respectively. All eicosanoids are secreted and thereby exert autocrine and/or paracrine functions. **d** Neutral lipolysis. Hydrolysis of triacylglycerols (TAG) to FA and diacyglycerol (DAG) is driven by the adipose triglyceride lipase (ATGL), while the DAG breakdown, producing monoacylglycerol (MAG) and FA, is catalyzed by the hormone-sensitive lipase (HSL). Finally, the monoacylglycerol lipase (MAGL) hydrolyses MAG into glycerol and FA. SSO sulfo-N-succinimidyl oleate, ETC electron transport chain, MUFA monounsaturated FA, lysoPL lysophospholipid, 5-HPETE 5-hydroperoxyeicosatetraenoic acid. LPCAT Lysophosphatidylcholine acyltransferase, GPAT glycerol-3-phosphate acyltransferase, ACSS2 ACS short-chain family member 2, TOFA 5-Tetradecyloxy-2-furoic acid, NDGA nordihydroguaiaretic acid, PTGES Prostaglandin E synthase, 15-PGDH 15-hydroxyprostaglandin dehydrogenase. Indirect and direct chemical reactions are illustrated by dotted and solid arrows, respectively.

FAs are either provided by diet or directly synthetized through the de novo synthesis pathway (Fig. 1). They are used as energetic sources to fuel tricarboxylic acid (TCA) cycle or as substrates for the synthesis of complex lipids, including structural lipids (e.g., phospholipids (PLs), sphingolipids) and intra- or extracellular signaling molecules (e.g., lysophospholipids, prostaglandins, leukotrienes) [1, 2] (Fig. 1). Different membrane-associated proteins are involved in the uptake of exogenous FAs by cancer cells from their environment, such as FA translocase (FAT or CD36), and FA transport protein (FATP) or in the intracellular transport of FAs, such as plasma membrane FA-binding protein (FABP) [7]. Nieman et al. have demonstrated that, during early stages of metastatic cascade, FABP4 promotes detachment and migration of cancer cells through the ECM towards the vasculature and is the core of the dialog between metastatic ovarian cancer (OvCa) cells and CAAs [18]. In this context, intracellular long-chain FA transport by FABP4, which is upregulated in patient’s omental metastases as compared to primary tumor, increases invasive capacities of OvCA cells [18, 19]. However, even if FABP4 is detected both in OvCA cells and CAAs, systemic treatments targeting tumor and host FABP4 are not more effective in decreasing metastatic nodules than host FABP4 knock-down [19, 20], highlighting the prominent role of FABP4 in TME [18–20]. In clinic, FABP4^high^ patients show poor overall and progression-free survival, and can be discriminated from FABP4^low^ patients by the lipid profile of OvCa cells, which is enriched in glycerolipids, PL and LysoPLs and in unsaturated and oxidized-FA lipid species [19]. In addition to FABP4, CD36 appears as an interesting metabolic target to limit metastatic progression in cancer. Indeed, its inhibition in OvCA cells restrains accumulation of LDs, cholesterol and lipid peroxidation products and diminishes their invasive and migratory capacities, as well as their adhesion on most ECM components of the peritoneum [21]. Consequently, intraperitoneal injection of CD36 KD cells gives rise to few metastases, and in an encouraging way, similar results are obtained after daily injections of neutralizing anti-CD36 antibody in wild-type (WT) OvCA xenograft models [21]. Inversely, CD36 overexpression in human oral carcinoma cells with low metastatic potential drives macro-metastasis in lymph nodes without potentiating primary tumor size [22]. This metastatic phenotype depends on increased expression of fatty acid oxidation (FAO)-related genes, and is boosted by a high-fat diet providing an enhanced supply of FAs to cancer cells. Interestingly, CD36-neutralizing antibody is equally effective on metastatic regression in intravenously-inoculated melanoma, oral carcinoma and mammary gland cancer mouse models [22]. Finally, a positive correlation between CD36 and FABP expression and EMT markers is observed in hepatocellular carcinoma (HCC) patients suffering of obesity [23]. Likewise, supplementation with palmitic or oleic acid improves HCC cell migration and activates the TGF-β and Wnt signaling pathways, both responsible of EMT program [23]. However, whether these effects are truly dependent on CD36-mediated FA uptake, still remains to be determined. Together, these studies provide evidences that FA transport is intimately connected to metastatic progression, at least in some cancers.

#### Catabolic pathways promoting metastasis

##### Fatty acid oxidation (FAO)

The initial substrates for FAO are acyl-CoAs, which result from the esterification of FAs by acyl-CoA synthetases (ACS) (Fig. 1). The resulting acyl-CoAs, which cannot be exported from cells, are then broken down into acetyl-CoA molecules, whose number depends upon the carbon chain length of the acyl-CoA being oxidized. Finally, FAO-derived acetyl-CoA is further oxidized through the TCA cycle, which in cooperation to oxidative phosphorylation, ultimately generates ATP and reducing equivalents, NADH and FADH_2_, to support redox homeostasis.

It is increasingly evident that, besides its undisputed role in tumor growth promotion [6, 7, 13], FAO is essential to support survival of ECM-detached cancer cells and their establishment in the metastatic niche [24, 25]. Indeed, in condition of loss of attachment, melanoma cells over-activate FAO to maintain high levels of NADPH and thereby prevent ROS-induced cell death [24]. Mechanistically, the FAO rate-limiting enzyme (i.e., mitochondrial trifunctional protein (TPβ)) (Fig. 1) is constitutively activated by the binding of phosphorylated Nur77 in the mitochondria. Hence, knockdown of TPβ or Nur77 reduces the number of circulating tumor cells and consequently the risk of lung colonization by these cells [24]. Interestingly, this is validated in patients with metastatic melanoma, who show higher Nur77 levels in metastases than in primary tumors [24]. Therefore, disrupting Nur77-TPβ interaction may constitute a therapeutic strategy for these metastatic patients. Another study demonstrated that melanoma cells undergo a metabolic shift from glucose/glutamine oxidation in primary tumors towards FAO in lymph node metastases to meet their bioenergetic needs [25]. Indeed, the higher increase in FAO gene transcription in lymph node-metastasis as compared to primary tumor and lung metastases results from activation of YAP signaling mediated by the binding of bile acids on vitamin D receptors [25]. Hence, administration of bile acids, generated from cholesterol in a CYP7A1 manner, promotes the growth of lymph node-metastatic melanoma, while depletion of CYP7A1 has an opposite effect [25]. Therefore, targeting YAP and/or CYP7A1 may be interesting therapeutic options to prevent the risk of cancer cell dissemination towards lymph nodes.

##### Neutral lipolysis

Triacylglycerols (TAG) constitute the storage form of excess intracellular FAs in LDs [26]. Their hydrolytic breakdown by 3 consecutive cytosolic lipases, i.e., neutral lipolysis, gives rise to FAs and glycerol (Fig. 1) [27]. Several studies showed the impact of blocking this metabolic route on metastasis formation, either directly by acting on lipolysis-mediated enzymes (e.g., monoacylglycerol lipase (MAGL) and hormone-sensitive lipase (HSL)), or indirectly on long-chain ACS (ACSL), promoting long-chain FA activation [28–30] (Fig. 1). Indeed, enhanced MAGL activity is a common feature of several aggressive cancers (i.e., melanoma, breast cancer (BC), OvCA) [28]. Its genetic or pharmacological inhibition leads to an accumulation of monoacylglycerols and subsequent reduction in FAs. These metabolic changes are accompanied by reduced tumorigenic capacities both in vitro and in vivo, which are fully rescued by supplementation in saturated fatty acids or high-fat diet, respectively [28]. Also in aggressive triple-negative breast cancer (TNBC), blocking the increased lipolysis induced by the cleaved-form of CUB-domain containing protein 1 (CDCP1), a feature also found in several other cancers (e.g., ovarian, lung, colon, prostate, and pancreatic cancers), decreases the metastatic abilities of TNBC cells in vitro and in vivo [30]. Mechanistically, disruption of CDCP1*-*ACSL interaction promotes activation of long-chain-FAs, and their subsequent degradation through the FAO and TCA cycle [30]. On the contrary, in Kras-driven pancreatic ductal adenocarcinoma (PDAC), HSL is down-regulated as compared to normal pancreas, and the subsequent decrease in HSL-induced lipolysis promotes the metastatic potential of mutated-KRAS PDAC cells [29]. This pro-metastatic phenotype is rescued by oleic acid supplementation, which restores intracellular LD pool. Interestingly, oleic acid primes PDAC cells to ECM degradation, invadopodia formation, as well as to migration and invasion. Consequently, HSL-expressing Kras^G12D^ PDAC cells are unable to form metastases neither primary tumor as compared to control Kras^G12D^ PDAC cells [29]. Importantly, these data highlight the therapeutic benefits of direct and indirect targeting of lipolysis on metastatic progression, especially for aggressive cancers.

#### Activated anabolic pathways supporting metastasis

##### De novo lipogenesis (DNL)

DNL, also known as de novo FA synthesis, is the metabolic pathway by which FAs are generated from different carbon sources (Fig. 1). In some cancers, this anabolic process is activated [4, 7, 31], and acetyl-CoA, produced from glucose catabolism in well-oxygenated condition or from alternative carbon sources, glutamine or acetate, under metabolic stress [32, 33], provides the substrate to DNL (i.e., citrate) (Fig. 1). Interestingly, in BC, non-small cell lung cancer (NSCLC) and prostate cancer (PCa), the relative contribution of de novo synthetized FAs from glucose and glutamine in the intracellular lipid pool is much lower than that of extracellular FAs (5–35% vs 65–75%) [7]. However, even if a therapeutic advantage of interrupting DNL on tumor growth has been demonstrated in several preclinical cancer models, the clinical development of more efficient, selective and safe DNL inhibitors is needed [34, 35]. Importantly, two recent studies showed that basal-like and HER2^+^ BC cells have a higher DNL rate when they grow in brain metastatic site rather than in primary one, and the DNL signature, characterized by increased cholesterol species and structural lipids, governs their ability to metastasize exclusively in the brain [36, 37]. These results are in accordance with clinic findings showing an up-regulation of *SREBF1* and *FASN* in brain metastasis relative to extra-cranial metastasis or matched-primary tumors in metastatic BC patients [36, 37]. Interestingly, *SREBF1*-depleted BC cells still able to seed but not to expand in brain microenvironment [36]. Similarly, the brain-permeable FAS inhibitor (i.e., BI99179) (Fig. 1) limits the growth of BC metastases in the brain, but does not slow primary tumor growth [37]. In the context of *Pten* null PCa where DNL contributes to the increase of TAG and PL pools, targeting of the SREBP-mediated lipogenic program using fatostatin also impedes distant lymph node metastasis, as well as primary tumor growth by decreasing the frequency of mitotic cancer cells and activating apoptosis [38]. Thus, these findings demonstrate that enhanced DNL pathway is a common feature of BC and PCa metastasis that can be targeted to slow down disease progression and expansion.

##### Prostaglandin and leukotriene synthesis

The released arachidonic acid (ARA) from membrane PLs, by the cytosolic phospholipase A2 alpha (cPLA2α), is further metabolized by 2 rate-limiting enzymes into active metabolites, namely eicosanoids (Fig. 1). The cyclooxygenases (COX) generate prostaglandins (PG), prostacyclins and thromboxanes, while the lipoxygenases (LOX) are responsible of leukotriene (LT) and hydroxyeicosatetraenoic acid (HETE) production [39] (Fig. 1). Once secreted, they mediate tumor promotion and progression by acting on producer or neighboring cells in an autocrine or paracrine manner, respectively [39] (Fig. 3). Regarding lung cancer, PGD_2_ to PGF_2_ are synthetized by both lung cancer cells and TME cells, while LTB_4_ to LTE_4_ are uniquely produced by tumor-associated-neutrophils (TANs) and macrophages (TAMs). A pro-metastatic role of cPLA2α and its end-products (PGE_2_, LTB_4_) has been demonstrated in several cancer models, including lung cancer, TNBC, HCC, and colorectal cancer (CRC) [40–43]. For example, cPLA2α knock-down in high cPLA2α-expressing TNBC cells with increased metastatic capacities, delays TNBC tumorigenesis and strongly decreases the number of lung metastasis nodules [42]. Importantly, in TNBC patients, elevated cPLA2α levels are correlated with tumor aggressiveness, and poor overall- and free-disease survival [42]. In accordance with these findings, administration of PGE_2_ potentiates the invasive capacities of CRC cells and their ability to form liver and lung metastases in preclinical models [41]. In contrast, the blockade of PGE synthase (PTGES), downstream of COX (Fig. 1), inhibits metastatic lung tumor growth by decreasing abundance of immunosuppressive cells, such as myeloid-derived suppressor cells (MDSCs) and TAMs, and restoring the anti-tumoral immune populations (e.g., CD8^+^ T and natural killer (NK) cells) [43]. In regards with TME-derived eicosanoids, it has been shown that LTB_4_ and PGE_2_ exert a crucial role in establishing the pre-metastatic niche in breast and liver cancers [44]. For example, the CD11b^+^Ly6G^+^ neutrophils’ enrichment of lung metastatic TME, observed before mammary cancer cells have infiltrated the tissue, and the subsequent increase in neutrophil-derived LTB_4_-E_4_ favor the high metastatic potential of mammary tumor cells [44]. Hence, specific-inhibition of LT synthesis with Alox5 inhibitor, suppresses neutrophil pro-metastatic activity, and thereby decreases metastatic incidence [44]. Interestingly, ALOX5 inhibitor reduces also the number of lung metastatic foci arising from HCC cells, similarly to the selective depletion of alveolar macrophages which are the predominant cells that produce LTB_4_ in this model [45]. In addition to mediate a dialog between tumor cells and TME cells, PGE_2_ is also involved in the crosstalk between lung resident mesenchymal cells and metastasis-infiltrating neutrophils, which stimulates mammary metastatic dissemination [46]. Indeed, lung resident mesenchymal cells increase lipid uptake and TAG storage in metastasis-infiltrating neutrophils through PGE_2_-dependent and -independent mechanisms [46]. These neutrophils produce TAG-loaded extracellular vesicles, which through a macropinocytic uptake, increase cell proliferation and metastatic colonization capacities of tumor cells in metastatic mammary cancer models [46]. In addition, in the context of bone metastases caused by mammary cancer cell dissemination, increased ARA secretion by osteoclasts and decreased lysophosphatidylcholine (LPC) secretion, both synergistically support proliferation and pro-metastatic features of tumor cells [47]. Therefore, treatment combining an inhibitor of ARA-derived PG and LT with LPC supplementation greatly reduces the mammary metastatic incidence and spreading in bone environment. Altogether, these studies clearly highlight the autocrine and paracrine pro-metastatic functions of tumor- and stromal-derived ARA metabolism, and identify cPLA2α and PTGES enzymes, and the PGE_2_ and LTB_4_-E_4_ end products, as key players in the promotion of mammary cancer, HCC and CRC seeding and colonization in lung and/or bone environment.

##### Synthesis of cholesterol and its derivatives

**Fig. 2:**
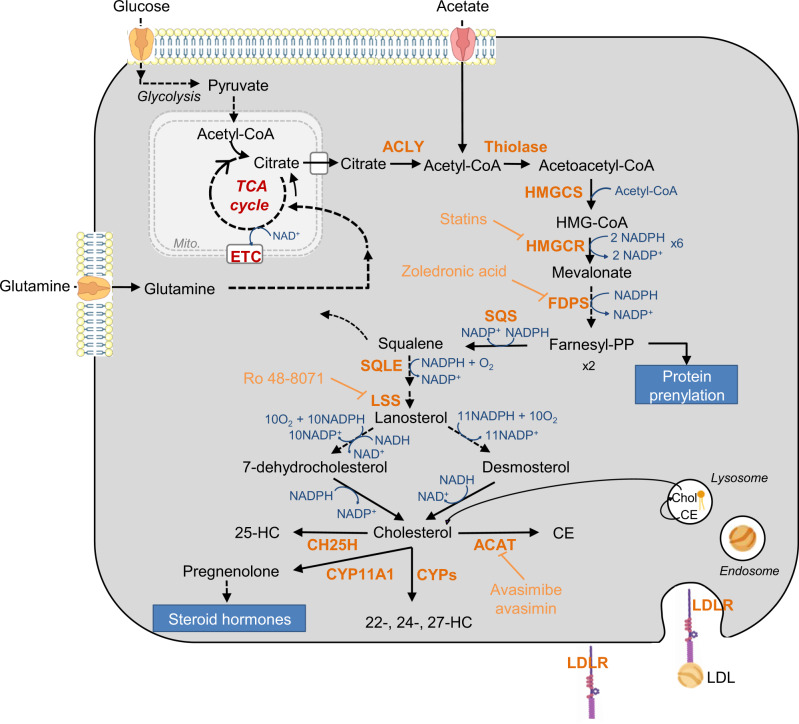
Schematic representation of key steps of the mevalonate (MVA) pathway leading to sterol and non-sterol isoprenoid synthesis. MVA is produced from acetyl-CoA, through reactions that are catalyzed by the acetoacetyl-CoA thiolase (Thiolase), HMG-CoA synthase 1 (HMGCS1) and HMGCR enzymes. MVA is converted into farnesyl-pyrophosphate (FPP), which with the geranylgeranyl-PP are responsible of protein prenylation. FPP molecules can also be condensed by the squalene synthase (SQS) to produce squalene. Then, squalene is oxidized and cyclized to lanosterol by the squalene epoxidase (SQLE) and lanosterol synthase (LSS), respectively. Finally, lanosterol is converted to cholesterol by 19 oxygen-based reactions through the Kandutsch-Russell or the Bloch pathway. De novo synthetized cholesterol and diet-derived cholesterol taken up through the LDL receptor (LDLR)-mediated endocytosis can give rise to cholesteryl esters, hydroxycholesterol (HC) or steroid hormones. HMG-CoA 3-Hydroxy-3-methylglutaryl-coenzyme A, ETC electron transport chain, TCA tricarboxylic acid, CE cholesteryl ester, Chol. cholesterol, 22-, 24-, 25- and 27-HC 22-, 24-, 25- and 27-hydroxycholesterol, CH25H Cholesterol 25-hydroxylase, CYP11A1 cytochrome P450 family 11 subfamily A member 1, CYPs cytochrome P450 enzymes, LDL low density lipoprotein. Indirect and direct chemical reactions are illustrated by dotted and solid arrows, respectively.

Cholesterol - Cholesterol, provided by diet or synthetized through the mevalonate (MVA) pathway, is part of the composition of cell membranes and serves as backbone of steroid hormones and bile acids [48–50] (Fig. 2). Moreover, its oxidation leads to oxysterol production [51]. Its synthesis from acetyl-CoA is an aerobic process with high energy cost and electron donor requirements (Fig. 2). Composed of 37 reactions, this pathway is governed by two rate-limiting enzymes, the 3-hydroxy-3-methylglutaryl-CoA reductase (HMGCR) and the squalene epoxidase (SQLE) (Fig. 2). In specific preclinical cancer models, blocking the over-activation of MVA pathway by targeting HMGCR with statins, has consistent anti-tumoral effects, however their clinical benefits remain disparate [8, 52, 53]. Likewise, SQLE inhibitors, due to their severe adverse effects, have been stopped in clinic [54], therefore the development of more selective and potent inhibitors needs to be pursued.

In metastatic context, using an in silico approach, Zhao and co-workers identified two interesting FDA-approved drugs (i.e., a PG agonist and a dopamine receptor antagonist), which are associated with an EMT gene signature [55]. Administered as monotherapy, they impair metastatic incidence without affecting growth of parental tumors in mammary gland mouse model [55]. This effect due to membrane cholesterol-loading, results from a defect in cholesterol efflux, which leads to reduced membrane flexibility and impairment of cell migration, mammosphere formation and EMT in several BC cell lines [55]. Consistently, a high intracellular cholesterol content is considered as a favorable prognosis factor in overall- and free-recurrence survival of HCC patients having undergone tumor resection [56]. Mechanistically, cholesterol abundance causes retention of CD44 in lipid rafts, and thereby disrupts its interaction with Ezrin and the actin cytoskeleton, which is crucial for HCC cell migration and metastatic spreading [56]. Inversely, the decreased intracellular cholesterol amount, caused by MVA pathway inhibition, is associated with acquisition of a metastatic phenotype by BC cells [57]. However, depending on the cancer type, the aberrant accumulation of cholesterol in membrane can have opposite effects. Indeed, in PCa cells, this event is associated with activation of EMT, cell migration and invasion [58]. In this model, EMT is mediated by stabilization of EGFR in cholesterol-rich plasma membrane domains (i.e., lipid rafts) and subsequent constitutive activation of the ERK1/2 cascade. Similarly, cholesterol-rich low-density lipoproteins (LDL) stimulates in vitro the migration and invasion abilities of PCa and PDAC cells, as well as expression of EMT markers [59]. Finally, the use of inhibitors targeting post-squalenic enzymes of the MVA pathway to limit metastatic disease, such as lanosterol synthase (LSS) or NAD(P)H steroid dehydrogenase-like (NSDHL) shows encouraging results. Indeed, LSS inhibition in vivo strongly hampers liver and lung metastatic burden of PDAC and colon carcinoma and extends the mice survival [60]. These effects result from a strong reduction of angiogenesis and of the migratory and adhesion capacities of endothelial cells [60]. Interestingly, blocking the NSDHL confers a more aggressive phenotype to PDAC (i.e., basal-like subtype), while decreasing tumor mass in Kras^G12D^/*Trp53* null mice [61]. This phenotype is explained by an activation of SREBP1, which in addition to stimulate MVA pathway gene transcription, transcriptionally activates the TGFβ-mediated EMT program [61]. However, further studies are necessary to determine whether the effects are specific of cholesterol itself or its derivatives (e.g., cholesteryl esters, oxysterols, etc.).

Cholesteryl esters (CEs)- To prevent cytotoxic excess of free cholesterol in cancer cells, cholesterol is locally esterified with long-chain FA-CoA by acyl-coenzyme A: cholesterol acyltransferases (ACATs) and stored in LDs [62] (Fig. 2). Interestingly, preventing CE accumulation, a feature shared by several types of tumors, including metastatic ones, decreases cancer aggressiveness [63–65]. For example, ACAT inhibitor dramatically impairs PCa cell migration capacities, and decreases PCa metastases incidence and growth through the Wnt/β-catenin signaling pathway [63]. Similar treatments, resulting in an excess of free cholesterol, provoke endoplasmic reticulum (ER) stress-induced apoptosis in PTEN-driven PDAC cells, decrease their migratory and invasive capacities as well as their propensity to grow in primary site and spread in lymph node and liver [64].

Oxysterols- Cholesterol is prone to enzymatically-induced oxidation or to auto-oxidation in oxidative stress conditions [66, 67]. The resulting products, generated by the addition of hydroxyl groups at different positions of the steroid skeleton, i.e., oxysterols (Fig. 2), exert pro-metastatic effects in distinct cancers, by acting as ligands of various nuclear receptors [68]. As an example, increased 27-hydroxycholesterol (HC) levels, induced by cholesterol-enriched diet in positive or negative estrogen receptor α mouse mammary cancer models, favor tumor recruitment of immunosuppressive cells (i.e., polymorphonuclear-neutrophils and γδ-T cells) at the expense of cytotoxic CD8^+^ T cell depletion [69]. As 27-HC acts mainly on host immune cells, its pro-metastatic effect extends to CRC, melanoma, PDAC and lung cancer [69]. Regarding the 25-HC, it enhances migration and invasion abilities of gastric and lung cancer cells, through the Toll-like receptor 2/NF-kB or LXR/IL-1B signaling pathways, respectively [70, 71]. In vivo, pre-treatment of gastric cancer cells with 25-HC potentiates their seeding and expansion in lung metastatic site [70]. To summarize, the host’s 25- and 27-HC confer metastatic capacities to cancer cells and activate recruitment of immunosuppressive cells at the tumor site, to ultimately lead to metastatic colonization and growth.

### Extrinsic and intrinsic lipid determinants and pathways in effector functions of TME cells

**Fig. 3:**
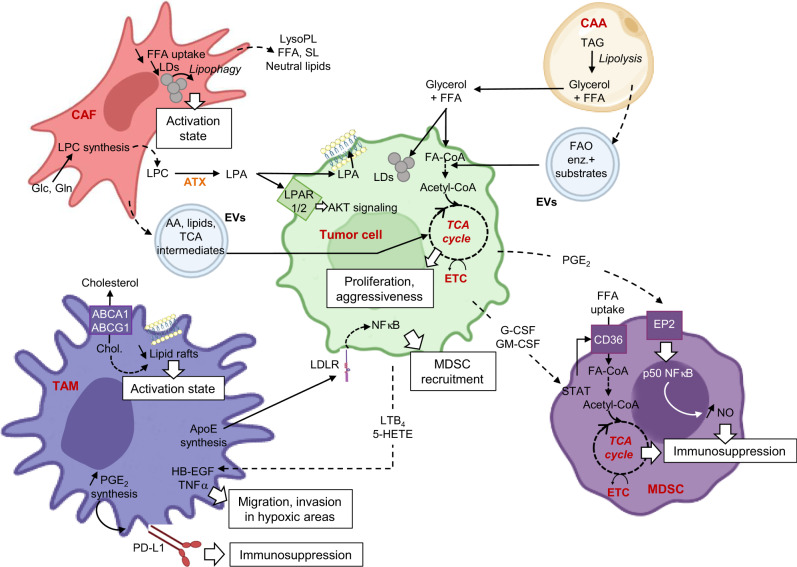
Non-exhaustive scheme of lipid exchanges between tumor cells and tumor microenvironment (TME) cells and lipid reprogramming of stromal cells, that activate their pro-tumoral features. Cancer-associated fibroblasts (CAFs) take up free fatty acid (FFAs), produce various lipids and together with cancer-associated adipocytes (CAAs) produce extracellular vesicles (EVs) that reach tumor cells to supply them with metabolic intermediates and enzymatic arsenal to activate metabolic pathways, such as fatty acid oxidation (FAO). CAAs are the seat of activated lipolysis, and the resulting FFAs once released in the TME can be taken up by tumoral cells. In tumor-associated macrophages (TAMs), the decreased in lipid rafts domains, resulting from increased cholesterol efflux, participate to TAMs activation. In these cells, prostaglandin E_2_ (PGE_2_) synthesis is also activated and contribute to the generation of an immunosuppressive tumor microenvironment. This latter is also promoted by myeloid-derived-suppressor cells (MDSCs), through the activation of FFA uptake and degradation and of the PGE_2_-PGE_2_ receptor 2 subtype (EP2) signaling pathway. Tumor cells are the recipients of various lipid species released in the TME by TME cells and are the mediators of an immunosuppressive TME via their release of lipids and cytokines. TAG triacylglycerides, TCA tricarboxylic acid, LPA lysophosphatidic acid, LPC lysophosphatidylcholine, ATX autotaxin, ETC electron transport chain, FA-CoA fatty acyl-Coenzyme A, LD lipid droplets, Gln glutamine, Glc glucose, SL sphingolipids, AA amino acids, lysoPL lysophospholipids, G-CSF granulocyte-colony stimulating factor, GM-CSF granulocyte macrophage colony stimulating factor, NO nitrogen oxide, 5-HETE 5-dydroxyeicosatetraenoic acid, LTB_4_ leukotriene B_4_, HB-EGF Heparin-binding EGF-like growth factor, TNFα tumor necrosis factor α, LDLR low-density lipoprotein receptor, PD-L1 programmed cell death-ligand 1, ABCA1 ATP Binding Cassette Subfamily A Member 1, ABCG1 ATP Binding Cassette Subfamily G Member 1. Indirect reaction is illustrated by dotted arrow and direct reaction by solid arrow.

The TME, which may account for up to 90% of the growing tumor mass in some cancers [72], is composed of tumor-infiltrating host non-malignant cells and ECM components [73]. The TME’s cellular components include CAFs, immune cells, CAAs and endothelial cells. Although stroma contribution to tumor cell growth and dissemination is well recognized, there is little knowledge in regards with the lipid-mediated dialog between both cell types and the contribution of cancer cells in lipid remodeling of TME cells, underlying the phenotypic switch from anti- to pro-tumoral functions (Fig. 3).

#### Cancer-associated fibroblasts (CAFs)

In PDAC, CAFs are the prominent stromal cells, and are composed of several subtypes showing distinct functional features in cancer progression and therapy resistance [74]. In pancreas, the pancreatic stellate cells, which are resident cells, can give rise upon tumor-derived activating signals to a CAF subtype [75]. Interestingly, the acquisition of CAF phenotype is characterized by a deep down-regulation of genes involved in lipid uptake and storage into LDs [10] (Fig. 3). This results in decreased LD content through lipophagy, and increased secretion of lysoPLs, free FAs (FFAs), sphingolipids and neutral lipids (Fig. 3). Moreover, CAFs from PDAC or PCa patients produce extracellular vesicles loaded of building blocks and metabolites, such as amino acids, FFAs and TCA intermediates, which are utilized by nutrient-deprived cancer cells to support their proliferation (Fig. 3). In FA–limiting conditions (i.e., low-lipid serum and FA desaturation inhibition), the CAF-secreted lipids restore the PDAC cell proliferation rate observed in non-depleted media [10]. Indeed, CAF-secreted LPC are either converted by autotaxin into lysophosphatidic acid, that binds to LPAR1/2 receptors and activates AKT signaling in tumor cells, or are directly taken up by PDAC cells to contribute to de novo membrane synthesis [10] (Fig. 3). Interestingly, the pharmacological targeting of autotaxin significantly reduces PDAC tumor growth, an effect that is accentuated by the co-transplantation of pancreatic stellate cells and cancer cells [10], highlighting the promoting role of lipid dialog between cancer and stromal cells in tumor growth. However, it remains to be determined whether this lipid phenotype is specific to pancreatic stellate cells-derived CAFs or a common feature of recently identified CAF subtypes (i.e., myofibroblastic, inflammatory and antigen-presenting CAFs) [76].

#### Cancer-associated adipocytes (CAAs)

Existence of a FFA-based metabolic symbiosis between CAAs and cancer cells, which facilitates tumor development and metastatic progression, has been originally described in the main OvCa metastatic site, as previously commented in this review [18]. Similar FFA-dependent dialog occurs between mesenteric adipocytes and the neighboring colon cancer cells, when they are in a nutrient-deprived TME [77]. In vivo, facilitating this metabolic exchange, by co-implantation of adipocytes and colon cancer cells, potentiates tumor growth. Indeed, adipocyte-derived FFAs support cancer cell survival upon glucose-starved condition by activation of FAO and autophagy to meet their energy demand (Fig. 3) [77]. In breast cancer, adipocytes also promote accumulation of LDs containing TAGs, cholesterol and CE [78]. Interestingly, BC cells cultivated with adipocyte-conditioned medium undergo a metabolic switch towards uncoupled mitochondrial FAO, and consequently rely on anaerobic glycolysis for their ATP production [78]. This shift is associated with increased EMT features and invasive capacities of BC cells, which are completely abrogated following inhibition of the ATGL-dependent lipolysis or CPT1-dependent FAO pathway [78]. Importantly, the same authors previously demonstrated that CAA-derived extracellular vesicles take part in the metabolic dialog between CAA and cancer cells as they transfer functional FAO enzymes and substrates to melanoma cells and this is associated with an increase of their aggressiveness, a phenotype reversed by FAO inhibitors [79, 80] (Fig. 3). Interestingly, CAA-derived extracellular vesicles from overweight or obese individuals gradually increase melanoma cell migration compared with those from lean individuals [79]. These findings were further validated in a melanoma obese mouse model submitted to a high fat diet [80]. Collectively these results shed the light on the role of CAA as donor of FFAs or -lipid-loaded extracellular vesicles and promoter of the metastatic potential of cancer cells.

#### Tumor-infiltrating immune cells

Tumor-infiltrating immune cells exert important functions in cancer progression and dissemination, as well as in treatment resistance [81]. The composition of the immune infiltrate varies between tumor types and subtypes, and between primary vs metastatic sites [82]. In most tumors, the anti-cancer immune response carried out by the cytotoxic lymphocyte subsets, CD8^+^ T cells and natural killers (NKs) is defective, and as a counterpart, immunosuppressive cells such as MDSCs, neutrophils, immune regulatory T (Treg) and M2-like macrophages are recruited to tumor site to aid tumor growth [81]. Understanding the metabolic reprogramming behind the switch from pro-tumoral to anti-tumoral immunity TME could help to elaborate new therapeutic strategies and improve gold-standard immunotherapy response.

##### Immunosuppressive cells

MDSCs- Their recruitment at the primary site by tumor- and host-secreted pro-inflammatory factors prevents the elimination of pre-malignant and malignant cells by suppressing T cell activation, and thereby promote tumor growth [83]. Acquisition of MDSC immunosuppressive functions depends on tumor-derived lipids, such as PGE_2,_ since treatment with agonists of PGE_2_ receptors (EP2) promotes the differentiation of bone marrow myeloid progenitors into suppressive CD11b^+^ Gr^+^ MDSCs, precursors of macrophages and neutrophils [83]. Mechanistically, it has been shown that the tumor-derived PGE_2_, by its binding to EP2 receptors, promotes nuclear accumulation of p50 NF-κB and NOS-mediated immunosuppression of monocytic MDSC subpopulation [84] (Fig. 3). Hence, the PGE_2_ - EP2 axis appears as a promising pathway to restore anti-tumoral immunity as demonstrated in fibrosarcoma and melanoma pre-clinical models [84, 85]. In the first model, blocking EP2 reprograms monocytic MDSC towards a NOS2^low^/TNFα^high^ phenotype and restores anti-tumoral efficacy of IFNγ [84], while in the second one, PGE_2_ synthesis inhibitor limits secretion of protumoral cytokines and growth factors by bone marrow-derived mononuclear cells and thereby stimulates tumor destruction by T cells [85]. Besides the PGE_2_/EP2-mediated immunosuppressive phenotype of MDSC, increased lipid uptake and their ultimate degradation into the TCA cycle and subsequent increase in OXPHOS activity are essential events for anti-tumoral functions of MDSCs [86]. Indeed, in Lewis lung tumor-bearing mice, this metabolic feature is driven by tumor-derived G-CSF and GM-CSF, that promote the CD36-mediated FA uptake in tumor-infiltrating MDSCs [87] (Fig. 3). Hence, in vivo treatment with FAO inhibitor blocks T-cell proliferation and IFNγ production and thereby delays tumor growth [86]. Similarly, implantation of Lewis lung cancer cells in systemic CD36 KO mice gives rise to smaller tumors than those of WT mice, and this anti-tumoral effect is completely counteracted by CD8^+^ T cells depletion [87]. Of note, similar results are obtained following colon cancer cell implantation [87]. These findings clearly highlight that uptake and catabolism of exogenous lipids could be a therapeutic lever to avoid MDSC-mediated immunosuppression.

Tumor-associated macrophages (TAMs) - With their precursors, TAMs constitute the most prominent cells of the myeloid infiltrate in several solid tumors [88, 89]. Although they are commonly classified in pro-inflammatory (M1) or anti-inflammatory macrophages (M2), which restrain or support tumor progression, respectively, a broad spectrum of intermediate M2 phenotypes with distinct functions has been described [88–90]. In aggressive hypoxic tumors, such as OvCa, TAM recruitment is mediated by tumor-derived lipid chemoattractants, like LTB_4_ and 5-HETE [91] (Fig. 3). Indeed, inhibition of their synthesis by Zileuton, a specific ALOX5 inhibitor, significantly reduces macrophage infiltrate in OvCa hypoxic areas and tumor growth [91] (Fig. 3). In PDAC, TAMs participate to the development of an immunosuppressive TME through secretion of Apolipoprotein E (APOE) [92], a core component of blood lipoproteins. The authors showed that macrophage-derived APOE, through its binding to LDL receptor (LDLR) and activation of NF-κB signaling, activates Cxcl1 and Cxcl5 production in PDAC cells, which drive the recruitment of myeloid cells and the suppression of tumor-infiltrating CD8^+^ T cells [92]. Consequently, PDAC mouse models deprived of systemic *ApoE* develop small tumors with a high apoptotic index, few immunosuppressive cells and increased number of cytotoxic CD8^+^ T cells [92]. These findings are in accordance with the clinic, since high levels of APOE in blood of patient’s PDAC are correlated with poor survival [92]. Other reprogrammed lipid pathways drive the immunosuppressive functions of TAM, such as cholesterol efflux [93], ARA-derived eicosanoid synthesis [94, 95] or DNL [96], depending on the cancer type. In aggressive OvCA mouse models, cholesterol efflux, through the ABCA1 and ABCG1 transporters, is enhanced in F4/80^+^ TAMs, leading to lipid raft depletion, defect in STAT6 and PI3K-mTORC2-AKT signaling which is correlated to increased IL4-dependent activation of macrophages [93] (Fig. 3). Consequently, myeloid-specific deletion of *Abca1/Abcg1* reverts the tumor-promoting functions of TAMs and thereby slows down OvCA progression [93]. In bladder tumor-bearing mice, tumor-infiltrating F4/80^+^ macrophages, as monocytic MDSCs, show high levels of PGE_2_-forming enzymes and cell-surface programmed cell death-ligand 1 (PD-L1) [94]. Hence, decreasing levels of PGE_2_ results in a reduction of PD-L1 expression in bladder tumors, and a subsequent abolishment of immunosuppressive TME [94]. In contrast, RCC-infiltrating TAMs synthetize 15(S)-HETE in a 15-LOX2 dependent manner, instead of PGE_2_ [95]. As a consequence, inhibition of LOX attenuates immunosuppressive functions of RCC-infiltrating TAM. Finally, the contribution of SREBP1-dependent DNL in the phenotype switch from M1 to M2 has been clearly demonstrated in melanoma and colorectal models [96]. Interestingly, this M2 metabolic feature can be lost following the inhibition of the immunosuppressive activity of Tregs on CD8^+^ T cells [96]. Hence, targeting Tregs to impair FA metabolism in M2-like TAM could be an interesting strategy to shift from a pro-tumoral towards an anti-tumoral TME and thereby limit tumor expansion and improve cancer immunotherapy.

CD4^+^ T cells - They comprise different subtypes, some of which exert pro-tumoral functions such as T-helper 2 (Th2) cells, or acquire an immunosuppressive status such as Treg cells [81]. In Treg cells, activation of DNL and cholesterol synthesis, appears essential for their proliferation and expression of suppressive molecules, such as CTLA-4 and ICOS [97]. Once infiltrated in tumors (i.e., NSCLC, melanoma and colon carcinoma), Tregs adapt to the TME’s nutrient availability, by shifting their metabolism from DNL to CD36-mediated FA uptake [98]. In this context, the Treg-specific depletion of *Cd36* attenuates OXPHOS, impairs mitochondrial fitness, decreases the NAD-to-NADH ratio, and activates the glycolytic activity of Treg cells that no longer survive under lactic acid-enriched TME [98]. These metabolic changes delay tumor growth and revert TME status, moving from immunosuppressive to anti-tumoral phenotype, while preserving peripheral immune homeostasis [98]. However, conflicting results were described in similar tumor-bearing mouse models, in which the intratumoral Treg proliferation was shown to depend from an increased FA synthesis rather than an enhanced FA uptake and abrogating FA synthesis leads to similar results [99, 100]. Indeed, in colon tumors, the Treg advantage is sustained by an enhanced glycolytic flux towards citrate, to promote FA synthesis rather than ATP production through OXPHOS [99]. Interestingly, Treg-specific targeting of SREBP activity or FAS, leads to a rapid and complete tumor regression through activation of distinct mechanisms [100]. While *Fasn* depletion impairs TCR-induced maturation of Tregs, blocking SREBP activity inhibits the geranylgeranylation of PD-1, a post-translational modification, and thereby the tumor-selective induction of PD-1 expression on Treg cells [100]. Further studies are required to better understand the contribution of each pathway in Treg immunosuppressive functions and to evaluate the efficacy of combination therapies as compared to monotherapies.

In regard of immunosuppressive Th2 cells, the cell-specific targeting of *Cyp11a1*, encoding for an enzyme converting cholesterol into pregnenolone, the precursor of all other steroid hormones (Fig. 3), restricts primary and metastatic melanoma and mammary tumor growth [101]. This highlights a role for Cyp11a1 in the anti-tumor immunity response and shows the direct involvement of steroidogenesis in promoting immunosuppressive activity of tumor-infiltrating Th2 cells.

Dendritic cells (DCs) - They process and present tumor-derived antigens to naïve T cells, however this anti-tumoral status can be reversed by immunosuppressive lipid signals from tumor cells. For example, CCR7-dependent maturation of DCs, as well as their migration to draining lymph nodes where they exert their anti-tumor immune responses, are inhibited by tumor-derived oxysterols [102]. Hence, inactivation of oxysterols by inducing its sulfurylation by the sulfotransferase 2B1b enzyme, in lymphoma, prostate or lung tumors promotes tumor rejection and extends mice survival. The immunosuppressive status of tumor-infiltrating DCs is also promoted by *FASN*^high^ OvCA ascites, which leads to abnormal lipid accumulation and subsequent dampening of DC ability to present antigens and activate effector T cells [103]. Consequently, the systemic FAS inhibition increases tumor-infiltrating T cells and restores the DC-driven anticancer immune response in OvCA-transplanted mice [103]. This lipid overload is also observed in tumor- and lymph node-infiltrating DCs from patients with NSLC, RCC or head and neck cancer, as compared to peripheral blood DCs [104]. A recent study demonstrated that the binding of oxidatively truncated TAGs to HSP70 in DCs, prevents the trafficking of the peptide-MHC class I complexes to cell surface, and thereby antigen presentation by DCs [105].

Tumor-associated neutrophils (TANs) - Emerging evidence showed that TANs, derived from granulocytic-MDSCs, are key actors in tumor establishment and metastasis development by favouring immune escape and/or neoangiogenesis [106]. Their recruitment in tumor is conditioned by tumor-derived lipids, such as oxysterols, as for example, 22-HC, produced and secreted by murine T cell lymphoma, that serves as chemoattractant for pro-tumoral neutrophils [107]. Disrupting TAN recruitment delays lymphoma and mesothelioma tumor growth, dampens the infiltrating-proangiogenic neutrophils and endothelial cells and enhances overall survival of tumor-bearing mice [107]. In pancreatic neuroendocrine tumors (PNETs), 24S-HC seems to be implicated in the recruitment of neutrophils close to hypoxic tumor cells [108]. Hence, specific activation of oxysterol sulfurylation in pancreatic β-islets delays PNET development by reducing tumor-infiltrating neutrophils and endothelial cells in neoplastic islets. In a context of chronic inflammation, promoting cancer emergence, as for example following exposure to air-borne pollutants, accelerated tumor progression in spontaneous lung cancer-bearing mice is associated with an increased neutrophil recruitment, an event promoted by the binding of LTB_4_ on its receptor on neutrophil cell-surface [109]. In addition to their involvement in tumor-recruitment of neutrophils, lipids participate also to the activation of neutrophils in spleen of several tumor-bearing mouse models (lymphoma, lung or colon carcinoma) [110]. The increase in ARA uptake and the subsequent COX2-dependent PGE_2_ synthesis enhance the expansion of activated neutrophils that suppress antigen-specific CD8^+^ T cell response [110]. Hence, inhibition of ARA uptake significantly delays tumor growth, and this effect is abrogated by CD8^+^ T cell depletion [110]. Importantly, TANs produce more PGE_2_ and LTB_4_ than resident alveolar macrophages in lung carcinoma-bearing mice, a production that is completely abrogated by systemic cPLA2α loss [111].

##### Anti-tumor immune cells

CD8^+^ T cells - They constitute the main effectors of anti-cancer immune response and their role is to destroy cancer cells by inducing apoptosis or by fusion of their secreted-lytic granules with cancer cell membrane [112]. Although their cytotoxic activity is lost in most tumors, strategies aiming to revitalize dysfunctional CD8^+^ T cells are of great interest to clinically combat cancer. Recently, Markosyan and co-workers identified the ephrin-A receptor 2/TGF-β/SMAD/PTGS2 axis as a key tumor-intrinsic driver of immunosuppressive TME in spontaneous PDAC mouse model [113]. Consequently, *Ptgs2*-depleted PDAC cells give rise to smaller tumors than wild-type cells, an effect that is completely abolished by T-cell depletion [113]. The immune TME of *Ptgs2*-depleted PDAC is enriched in CD3^+^ and CD8^+^ T cells, and in activated DCs, while Treg cells are fewer [113]. Importantly, combining systemic *Ptgs2* loss does not promote additional benefits on *Ptgs2-*depleted tumor growth and mice survival, suggesting that tumor-derived PGE_2_ is sufficient to drive T cell-dependent immunosuppression and thereby cancer progression [113]. As for PGE_2_-rich TME, a recent study showed that cholesterol-rich TME induces a loss of cytotoxic functions of intratumoral CD8^+^ T cells [114]. Indeed, increased cholesterol uptake stimulates the ER stress sensor, XBP1, which then increases expression of PD-1 immune checkpoints. Therefore, blocking XBP1 in CD8^+^ T cells or decreasing cholesterol synthesis in tumor cells restores the cytotoxic activity of tumor-infiltrating CD8^+^ T cells in lung tumor-bearing mice [114]. In addition to increase immune checkpoints in CD8^+^ T cells, cholesterol-rich TME promotes CD36 over-expression, which is associated with tumor progression and poor survival [115]. Specific-CD36 depletion in melanoma- or lung tumor-infiltrating CD8^+^ T cells increases their anti-tumoral potential by stimulating production of cytotoxic cytokines and inhibiting ferroptosis [115]. As CD36 promotes uptake and accumulation of oxidized-lipids and thereby T-cell dysfunctions, its systemic or CD8^+^ T cell-specific deletion slows down tumor growth, and rejuvenates antitumor functions of infiltrated CD8^+^ T cells by preventing oxidized LDL-dependent lipid peroxidation [116]. This dependency on FAs is even more required under metabolic stress (hypoxia ± glucose starvation), in order to meet their biomass and ATP needs and preserve their effector functions [117]. In this context, promoting FA degradation accelerates tumor regression in melanoma-bearing mice [117]. To summarize, anti-tumoral functions of CD8^+^ T cells rely not solely on lipid and cholesterol supply from the TME, but also from cholesteryl esters [118]. Therefore, specific-inhibition of cholesterol esterification in CD8^+^ T cells slows down melanoma and lung tumor growth and extends survival of tumor-bearing mice [118]. The increased CD8^+^ T cell response results from an enhanced membrane cholesterol content, which improves, through TCR micro-clusters and the signalosome, the degranulation and polarization of the cytolytic granules [118]. Interestingly, the authors obtained similar results with avasimibe, the ACAT inhibitor clinically approved for other diseases [118].

NK cells - They show potent cytotoxic activity against cancer cells through the release of perforin and granzymes close to target cells or by receptor-induced target cell apoptosis, and they can also modulate the effector response of additional immune cells [119, 120]. This latter NK function can be driven by melanoma-derived PGE_2_, which decreases viability and production of classical type 1 DC chemo-attractants by NK [121]. As a consequence, less DC1 are recruited to the tumor site and thereby the anti-tumor immune response is compromised [121]. Therefore, invalidating *Ptgs2* in BRAF^V600E^ melanoma could be an effective strategy to improve the NK-dependent recruitment and functions of DC1 in the cancer immune control.

### Overcoming cancer cell therapy resistance by targeting lipid or cholesterol pathways

Resistance to cancer treatment is responsible for most tumor relapse and cancer-related deaths [122, 123]. Emerging evidences support a key role of metabolic reprogramming in the adaptive response of cancer cells to combat drug-induced toxicity [12, 124, 125]. Therefore, inhibiting metabolic pathways to deal with the escape of cancer cells to gold standard chemo- or immunotherapies constitutes interesting combination treatments to improve patient clinical outcome. Here, we summarize the targeting of several lipid metabolic pathways that can be used to increase tumor sensitivity to chemotherapeutic agents or immunotherapies in preclinical models.

#### Targeting chemotherapy resistance

##### Fatty acid transporters

Targeting FA uptake or intracellular FA trafficking improves the chemotherapy effectiveness in metastatic OvCA and HER2^+^ mammary tumors, respectively [20, 126]. Indeed, small FABP4 inhibitor (i.e., BMS309403) increases the carboplatin response of OvCA cells isolated from non-responder patients, and in vivo, the drug combination has greater anti-metastatic effects than the single treatment [20]. Interestingly, CD36-mediated FA uptake becomes the main source of lipids over FA synthesis in mammary cancer during acquisition of HER2-targeted therapy resistance (i.e., lapatinib) [126]. Hence, blocking CD36 suppresses the growth of lapatinib-resistant tumors by promoting cell apoptosis, while it has no effect on lapatinib-sensitive tumors [126]. These findings encourage the development of combined therapeutic approaches in which inhibition of FA supply or transport together with gold standard chemotherapy would reverse chemoresistance of OvCA and HER2 mammary tumors.

##### Fatty acid oxidation

Targeting FAO is a therapeutic metabolic approach conceivable thanks to the knowledge acquired in metabolic reprogramming of several chemoresistant tumors, as leukemia, gastric cancer, CRC and melanoma [127–130]. Such metabolic targeting has been successful for cytarabine (Ara-C) resistant leukemic cells, which preferentially use FA over glucose to fuel the TCA cycle and support high mitochondrial OXPHOS status as compared to sensitive ones [128, 129]. Hence, blocking CPT1 with etomoxir provokes a shift from high to low OXPHOS status, which markedly enhances cytotoxic effects of Ara-C on resistant leukemic cells [129]. Similarly, in gastric and colorectal tumors, combination of oxaliplatin with the CPT inhibitor, perhexiline, improves their chemosensitiveness by increasing ROS-induced cell death, and provokes drastic tumor regression compared to what observed with oxaliplatin alone [130]. However, some tumors have the ability to constantly adapt their metabolism to counteract drug-anti-tumoral effects. For example, the BRAF^V600E^ melanoma cells increase their CD36 cell-surface levels and PPARα-dependent FAO to overcome the MAPK inhibitor effects [127]. In this case, CPT1 inhibitor is needed to limit FAO, as well as glycolytic inhibitors to avoid a second metabolic adaptation (i.e., glycolytic shift) and secure the therapeutic benefit of MAPK inhibitors [127].

##### De novo lipogenesis

In solid tumors with DNL dependency, targeting DNL-driven enzymes, such as ACC, FAS and SCD1 (Fig. 1), increases the chemotherapy response [131–135]. For example, treatment of doxorubicin-resistant PCa cells with the ACC inhibitor, soraphen, potentiates their chemosensitivity by increasing unsaturated FA incorporation into membrane phospholipids, which renders PCa cells more susceptible to lipid peroxidation, and more prone to doxorubicin entry through passive diffusion [132]. In highly aggressive tumors, such as PDAC, a correlation between FAS^high^ in patients treated with gemcitabine (GEM) and their overall survival has been clearly established [133]. Interestingly, sequential GEM and FAS inhibitor (i.e., orlistat) treatments induce synergistic anti-proliferative effects on resistant PDAC cells which result from decreased stemness and self-renewal capacity of PDAC cells and enhanced ER-induced apoptosis [133]. Similarly, orlistat is a more effective combined-treatment to significantly delay cisplatin-resistant OvCA growth than chemotherapy alone [131]. In clinic, the most advanced FAS inhibitor reported to date is TVB-2640. Combined to taxane in advanced cancers, it shows promising safety profile in first-in-human study, therefore, a phase II trial is ongoing [134]. Finally, in sorafenib-resistant HCC tumors, disruption of over-activated monounsaturated FA synthesis with SCD1 inhibitor (i.e., SSI-4) enhances sorafenib-induced tumor regression via an activation of ER-stress response [135].

##### Lipid droplet biogenesis

Aberrant accumulation of LDs is also positively correlated with chemoresistance in solid cancers, such as CRC, OvCA and cervical tumors [136, 137]. Indeed, in CRC cells, the increased in LD production mediated by lysophosphatidylcholine acyltransferase 2 confers resistance to 5-fluorouracil combined with oxaliplatin, by preventing ER stress-induced apoptosis and reducing the tumor-infiltrating CD8^+^ T cells [136]. Hence, lysophosphatidylcholine acyltransferase 2 or LD biogenesis inhibitors significantly improves tumor regression induced by the dual chemotherapy and mice survival. Similar results have been described for ovarian and cervical tumor-bearing mice treated with carboplatin or paclitaxel, respectively, plus an inhibitor of the glycolytic enzyme, PFKFB3 [137]. This latter indirectly blocks LD biogenesis and lipophagy.

##### Prostaglandin E_2_ metabolism

Targeting COX2 or PGE_2_ receptors has beneficial effects on progression of multiple cancers [138–142]. For example, in cisplatin-resistant OvCA cells, overexpression of PGE_2_ receptor, EP3, constitutes an interesting target to counteract chemoresistance, as its genetic silencing enhances drug-induced apoptosis, and decreases tumoral proliferation and vascularization [138]. In NSCLC cells, cisplatin, adriamycin or GEM induces COX2 expression, which in turn inhibits drug-induced cell apoptosis by activating the anti-apoptotic protein BCL2 [139]. Consequently, COX2 inhibitor reverses the cisplatin-resistant phenotype of NSCLC in vivo [139]. As mechanisms of cisplatin-induced resistance are similar in gastric cancers, they are also overcame by COX2 inhibitor [140]. In the context of tumor recurrence associated with chemoresistance, it has been shown that elevated COX2 and secreted-PGE_2_ levels favor urothelial carcinoma repopulation by CK14^+^ cancer cells following chemotherapy-induced apoptosis [141]. Hence, combined treatment with COX2 inhibitor, like celecoxib or low-dose aspirin, with GEM-cisplatin attenuates primary and metastatic growth of resistant-urothelial carcinoma and the acquired-chemoresistance [141]. Finally, in regards of acute myeloid leukemia, cancer cells drive the synthesis and release of PGE_2_ by mesenchymal stromal cells, which in turn protect them from Ara-C-induced apoptosis [142]. Consequently, interrupting this PGE_2_-based dialog enhances the anti-tumoral drug-efficacy and mice survival as compared to Ara-C alone [142].

##### Cholesterol metabolism

In cancer cells, modulation of intracellular cholesterol amount and distribution, by acting on LDL uptake, MVA pathway, cholesterol esterification, or isoprenoid-mediated signaling pathway, constitute effective treatments to overcome the poor therapeutic response of solid tumors [60, 143–148]. In PDAC, interrupting the LDL-LDLR internalization provokes deep changes in free vs esterified-cholesterol content, which are accentuated upon GEM treatment in vivo [143]. Moreover, CE content is higher in GEM-resistant PDAC cells than in its sensitive parental counterparts [144]. Hence, limiting CE accumulation, by targeting LDLR or ACAT (Fig. 2), increases the in vivo GEM efficacy on PDAC growth [143, 144]. Aberrant cholesterol levels in the mitochondria fraction has also been reported in patient-derived HCC as compared to healthy liver [145]. In this context, inhibition of MVA pathway at a pre- or post-squalenic step (HMGCR and SQS, respectively) (Fig. 2), decreases the mitochondrial cholesterol content, potentiates the HCC cell response to mitochondria-targeting drugs or to doxorubicin [145]. Specifically, in cancers with over-activated MVA pathway, notably gallbladder and breast cancers, use HMGCR inhibitors, such as statins, sensitizes tumor cells to chemo- or HER2-targeted therapies, respectively, and reduces tumor growth and prolong survival time of gallbladder cancer-bearing mice [146, 147]. Similarly, treatment with lipophilic statin or zoledronic acid of resistant-BC cells to HER2-targeted therapy leads to growth inhibition and cell apoptosis [147]. Finally, inhibitors targeting post-squalenic enzymes, such as LSS or testis meiosis-activating sterol/sterol C4-methyl oxidase–like (SC4MOL) (Fig. 2), show promising synergistic effects when combined to chemo- or EGFR-targeted-therapies in several cancers [60, 148]. Interestingly, the latter drug combination effect results from an unexpected role of SC4MOL in promoting vesicular trafficking of EGFR towards the lysosome, and its subsequent degradation [148]. These findings provide evidence that reducing intracellular cholesterol content or isoprenoid-mediated signaling pathway are promising metabolic approaches to alleviate the chemoresistance.

#### Targeting immunotherapy resistance

##### Uptake, oxidation and synthesis of fatty acids

Inhibition of lipid metabolic pathways that are over-activated in immunosuppressive cells and involved in the tumor immune escape are strategies of choice to block immunotherapy resistance [96, 98, 149]. In melanoma, infiltrating-Treg cells suppress CD8^+^ T cell-derived IFNγ, which results in increased SREBP1-mediated FA synthesis in M2-like TAM supporting their immunosuppressive functions [96]. Hence, combination of SREBP inhibitor (i.e., fatostatin) with anti-PD-1 reduces tumor growth and prolong survival of melanoma-bearing mice by decreasing M2-like TAM infiltrate and by stimulating CD8^+^ T cell reinvigoration [96]. Similarly, the combined use of anti-CD36 antibody to inhibit the Treg-specific up-regulation of CD36 with anti-PD-1 treatment strengthens the anti-tumor response induced by the anti-CD36 antibody alone without causing any autoimmunity [98]. In Braf^V600E^-*Pten*^−/−^ melanoma, tolerization of DCs within the TME also contributes to immunotherapy resistance of this tumor. This process implies a metabolic shift from glycolysis towards CPT1A-driven FAO in DCs and subsequent defective activation of effector T cells while the differentiation of Treg cell is promoted [149]. Hence, blockade of CPT1A with etomoxir combined with anti-PD-1 antibodies suppresses tumor growth, and this effect is correlated to enhanced CD8^+^ T cell infiltrate and activated CD8^+^ T cell response. An alternative strategy to improve cancer immunotherapies is to restore or preserve the CD8^+^ T cell anti-tumoral response by re-activating metabolic pathways that are essentials for CD8^+^ T cell cytotoxic functions [115, 117]. For example, in Braf^V600E^ melanoma preclinical models, the progressive loss of CD8^+^ T cell response is partially restored through the over-activation of PPARα-driven FAO in an oxygen- and glucose-deprived TME, using PPARα agonist, like fenofibrate [117]. This treatment delays tumor progression, and enhances efficacy of PD-1 blockade [117]. Whether this recovering of tumor-infiltrating CD8^+^ T cell functions is able to slow down tumor progression over time remains to be determined. Another study demonstrated that TME’s poly-unsaturated FAs taken up by melanoma-infiltrating CD8^+^ T cells through CD36 provoke lipid peroxidation and ferroptosis, and thereby impair anti-tumor capacities [115]. Hence, when specific CD36 knock-down in CD8^+^ T cells is combined to anti-PD-1 antibodies, the T cell antitumor response is drastically improved, as well as survival of melanoma-bearing mice [115]. Collectively these findings highlight that various metabolism-based therapeutic approaches can be elaborated to alleviate TME’s immunosuppression or restore the killing functions of CD8^+^ T cells.

##### Prostaglandin synthesis

A complementary treatment to immunotherapy could be to prevent lipid-immunosuppressive mediators to reach their target cells. This appears feasible in lymphoma, lung and colon tumors in which PGE_2_ precursor starvation of tumor-infiltrating neutrophils dampens tumor growth [110]. Moreover, combination of this treatment with checkpoint inhibitors, like CTLA4 or PD-1 antibodies, increases CD8^+^ T cell infiltrate and thereby lung tumor regression in mice [110]. In several cancers, immunosuppression is driven by the secreted kynurenine, which is activated through the COX2-PGE_2_ axis [150]. Therefore, inhibiting COX2 blocks the tumor growth in OvCA-bearing mice that receive human allogeneic lymphocytes. This effect is associated with an increase in CD3^+^ and CD8^+^ tumor-infiltrating T-cells [150]. Targeting of COX2 is also a potential therapeutic pathway to circumvent immunotherapy failure in PDAC, as COX2 tumor-specific depletion combined to anti-PD-1 and agonist CD40 antibodies significantly decreases PDAC growth and extends survival of tumor-bearing mice, while monotherapies are mostly ineffective [113]. These benefic effects are completely abrogated by T-cell depletion. Finally, a last approach consists in forcing the conversion of PGE_2_ into its biologically inactive metabolite, by overexpressing the 15-PGDH enzyme. This leads to a significant reduction of RCC-infiltrating granulocytic MDSC cells, which is concomitant to an increase in T-cell tumor-recruitment [151]. As a consequence, the resistance of RCC to anti-PD-1 therapy is overcame, and a significant therapeutic advantage is observed in treated tumor-bearing mice overexpressing 15-PGDH, as compared to single treatments [151]. All these findings support a preponderant role of PGE_2_-dependant pathways in the promotion of immunosuppressive TME in solid tumors, which can be abrogated by several approaches, as previously described, to improve immunotherapies.

##### Mevalonate pathway

Disruption of cholesterol biosynthesis intermediates, such as farnesyl- and geranylgeranyl pyrophosphate (Fig. 2), using the FDA-approved zoledronic acid as agonist of γδ T cells, shows encouraging results for the treatment of immunotherapy resistant cancers [152, 153]. Indeed, its administration in combination with low-dose IL-2 in metastatic hormone refractory PCa patients increases the number of peripheral mature Vγ9Vδ2 T cells [152]. Similar results have been obtained in metastatic BC patients receiving the same combined therapy [153]. However, only patients who sustain robust Vγ9Vδ2 T cell number show a favorable clinical outcome [152, 153]. Likewise, high TME’s cholesterol levels in solid tumors provoke an up-regulation of immune checkpoints in CD8^+^ T cells, and their exhaustion [114]. Hence, inhibiting cholesterol-induced ER stress or reducing TME’s cholesterol reactivates CD8^+^ T cell effector functions in lung tumor-bearing mice [114]. However, an earlier study shows conflicting results. Indeed, blocking cholesterol esterification reverses the CD8^+^ T cell exhaustion phenotype in skin melanoma and lung cancer mouse models [118]. Mechanistically, aberrant cholesterol accumulation in plasma membrane enhances T-cell receptor clustering and signaling, which are responsible for T-cell activation and proliferation [118]. As a consequence, avasimibe and anti-PD-1 antibodies are more efficient than single treatments in controlling tumor progression and increasing survival of melanoma-bearing mice [118].

## Conclusion

Considerable progress towards understanding the role of lipid metabolic pathways in tumor progression has been made over the past decade and has enabled the development of several approaches using metabolic targeting to treat preclinical tumor models. These findings also highlight evidences that tumor cells and TME’s cells have distinct metabolic preferences and dependencies according to tumor type and tumor site (i.e., primary vs metastatic). Hence, successful treatments may require a combination of anti-metabolic drugs or anti-metabolic drug plus specific dietary regimen to counteract major obstacles, such as the tumor-specific metabolic flexibility and the metabolic heterogeneity of tumor and stroma compartments. Moreover, adaptive mechanisms of tumor resistance to chemotherapy and immunotherapy, can imply a reprogramming of lipid pathways and lipid dialog between cancer cells and TME cells that still needs to be explored. Therefore, deciphering the metabolic landscape specific from each TME’s subpopulation, and their dialog with neighboring tumor cells using emerging technologies, at the single cell scale, like RNASeq and metabolomics, coupled with spatial analysis will highlight how TME metabolically supports tumoral cells and thereby will accelerate the development of metabolism-based therapeutic strategies, which may greatly improve outcomes of cancer patients.

## Data Availability

All findings summarized in this manuscript come from articles cited in the reference list and available in the MEDLINE bibliographic database.
